# Host Plant Use by Competing Acacia-Ants: Mutualists Monopolize While Parasites Share Hosts

**DOI:** 10.1371/journal.pone.0037691

**Published:** 2012-05-25

**Authors:** Stefanie Kautz, Daniel J. Ballhorn, Johannes Kroiss, Steffen U. Pauls, Corrie S. Moreau, Sascha Eilmus, Erhard Strohm, Martin Heil

**Affiliations:** 1 Department of General Botany, Plant Ecology, University of Duisburg-Essen, FB BioGeo, Essen, Germany; 2 Department of Zoology, Field Museum of Natural History, Chicago, Illinois, United States of America; 3 Department of Biology, Portland State University, Portland, Oregon, United States of America; 4 Department of Zoology, University of Regensburg, Regensburg, Germany; 5 Research Group Insect Symbiosis, Max Planck Institute for Chemical Ecology, Jena, Germany; 6 Department of Entomology, University of Minnesota, St. Paul, Minnesota, United States of America; 7 Biodiversity and Climate Research Centre (BiK-F), Senckenberg Gesellschaft für Naturforschung, Frankfurt am Main, Germany; 8 Departamento de Ingeniería Genética, CINVESTAV, Irapuato, Guanajuato, Mexico; Royal Holloway University of London, United Kingdom

## Abstract

Protective ant-plant mutualisms that are exploited by non-defending parasitic ants represent prominent model systems for ecology and evolutionary biology. The mutualist *Pseudomyrmex ferrugineus* is an obligate plant-ant and fully depends on acacias for nesting space and food. The parasite *Pseudomyrmex gracilis* facultatively nests on acacias and uses host-derived food rewards but also external food sources. Integrative analyses of genetic microsatellite data, cuticular hydrocarbons and behavioral assays showed that an individual acacia might be inhabited by the workers of several *P. gracilis* queens, whereas one *P. ferrugineus* colony monopolizes one or more host trees. Despite these differences in social organization, neither of the species exhibited aggressive behavior among conspecific workers sharing a tree regardless of their relatedness. This lack of aggression corresponds to the high similarity of cuticular hydrocarbon profiles among ants living on the same tree. Host sharing by unrelated colonies, or the presence of several queens in a single colony are discussed as strategies by which parasite colonies could achieve the observed social organization. We argue that in ecological terms, the non-aggressive behavior of non-sibling *P. gracilis* workers — regardless of the route to achieve this social structure — enables this species to efficiently occupy and exploit a host plant. By contrast, single large and long-lived colonies of the mutualist *P. ferrugineus* monopolize individual host plants and defend them aggressively against invaders from other trees. Our findings highlight the necessity for using several methods in combination to fully understand how differing life history strategies affect social organization in ants.

## Introduction

Ant-plant protection mutualisms are excellent models to study ecology and evolution of mutualisms [1] as they often involve several partners on both sides of the interaction [2]. Even interactions among closely related species can differ in their degree of specificity and in the net fitness outcomes for the partners involved [3], [4]. Ant-plants (myrmecophytes) provide nesting space and/or food to defending ants [5]–[7]. In return, mutualistic ants aggressively protect their hosts from herbivores, pathogens and competing plants [8]. However, these interactions can be exploited by non-reciprocating ant species, which take advantage of the resources that are provided by the host plant without rendering any protective service [9]–[13].

In Mexico and Central America, several ant species of the genus *Pseudomyrmex* live in an obligate mutualism with acacia ant-plants. Both partners are highly adapted to this mutualism [12], [14]–[16]. The myrmecophytic acacias form hollow swollen thorns that serve as nesting space for the ants. Additionally, the plants provide nourishment in the form of extrafloral nectar as a food source for ant workers, and protein and lipid rich food (Beltian) bodies as nutrition for developing larvae. The mutualistic plant-ants are never found nesting apart from their hosts. They constantly patrol the plant surfaces and are extremely aggressive towards the plants' enemies. Equipped with a painful sting, the ants represent an effective indirect plant defense against a broad range of attackers [7], [8]. Myrmecophytes can be exploited by parasitic non-defending ant species that make use of plant-derived food resources and occupy nesting space but do not protect the plant. Acacias inhabited by such parasites suffer from severe herbivory resulting in loss of leaf area, dead shoot tips and retarded growth [12]. The parasitic species *Pseudomyrmex gracilis*, *Pseudomyrmex nigropilosus* and *Camponotus planatus* often occur sympatrically with mutualistic plant-ants [11], [12], [17].

Recent studies demonstrate that the ant mutualists in the acacia-*Pseudomyrmex* system have reduced digestive capacities and thus seem to depend completely on their plant hosts [14], [16]–[19]. Parasites inhabit acacia myrmecophytes and exploit plant-derived resources, but additionally use non-acacia food sources [16] as shown by stable isotope analyses [12]. These species reproduce at smaller colony sizes than the mutualists, a strategy that makes them even less dependent on the plants [11].

The ant association of acacia myrmecophytes and their ants inhabitants is a particularly well studied system, e.g., [19] and references therein. Despite the intensive research on this system, neither genetic factors nor behavior-mediating chemical factors (such as cuticular hydrocarbons) of the ant partners have been thoroughly analyzed. This is surprising since in-depth knowledge of these factors is essential to understand whether mutualism or parasitism is characterized by the evolution of specific traits (or combinations of traits) such as the genetic colony structure and chemically-mediated recognition of nestmates. In the present study, we applied a comparative approach integrating genetic microsatellite analyses, observations of behavior, and chemical analyses of cuticular hydrocarbon profiles.

In addition to affecting genetic characteristics of colony composition, the different strategies of mutualists and parasites have apparent implications for ant communication and behavior. The cues used by social insects to distinguish nestmates from foreign individuals are low-volatile chemicals present on the cuticle (usually hydrocarbons) [20], [21]. Colony members share a common chemical signature that is created by the admixture of individual profiles through allogrooming (i.e., social cleaning), trophallaxis (i.e., mouth to mouth feeding), and physical contact [22]. Individuals whose chemical signature deviates from the template are recognized as foreign and often attacked. Aggression between colonies is generally negatively correlated with overall hydrocarbon similarity, e.g., [23], [24]. Besides their function in nestmate recognition, cuticular hydrocarbon profiles are also species and caste specific [21]. Thus, a combination of chemical analyses of hydrocarbon profiles and behavioral experiments can provide important information on the social association among plant-ants that colonize a given host plant.

However, the cuticular hydrocarbon profile — and potentially the corresponding behavior — can be shaped by endogenous, genetic factors as well as exogenous, environmental factors e.g., [20], [25]. We combined behavioral and chemical data with genetic analyses of two sympatric ant species (a mutualist and parasite) that colonized ant-acacia species. This integrative study provides a robust approach for detecting colony boundaries while at the same time allows for evaluating the reliability of the different methods to investigate social organization in insect colonies. This is the first study to integrate these three approaches in two competing species of congeneric mutualistic and parasitic acacia-ants. Our findings highlight the necessity for combining these methods to fully understand how differing life history strategies shape genetic structure and communications of parasitic and mutualistic acacia-ants.

## Materials and Methods

### Ethics statement

As the ants and acacias used are wild species that are not protected and because all experiments were conducted on private grounds (with permission of the owners), no permits were required to perform the field experiments.

### Study Sites

**Figure 1:**
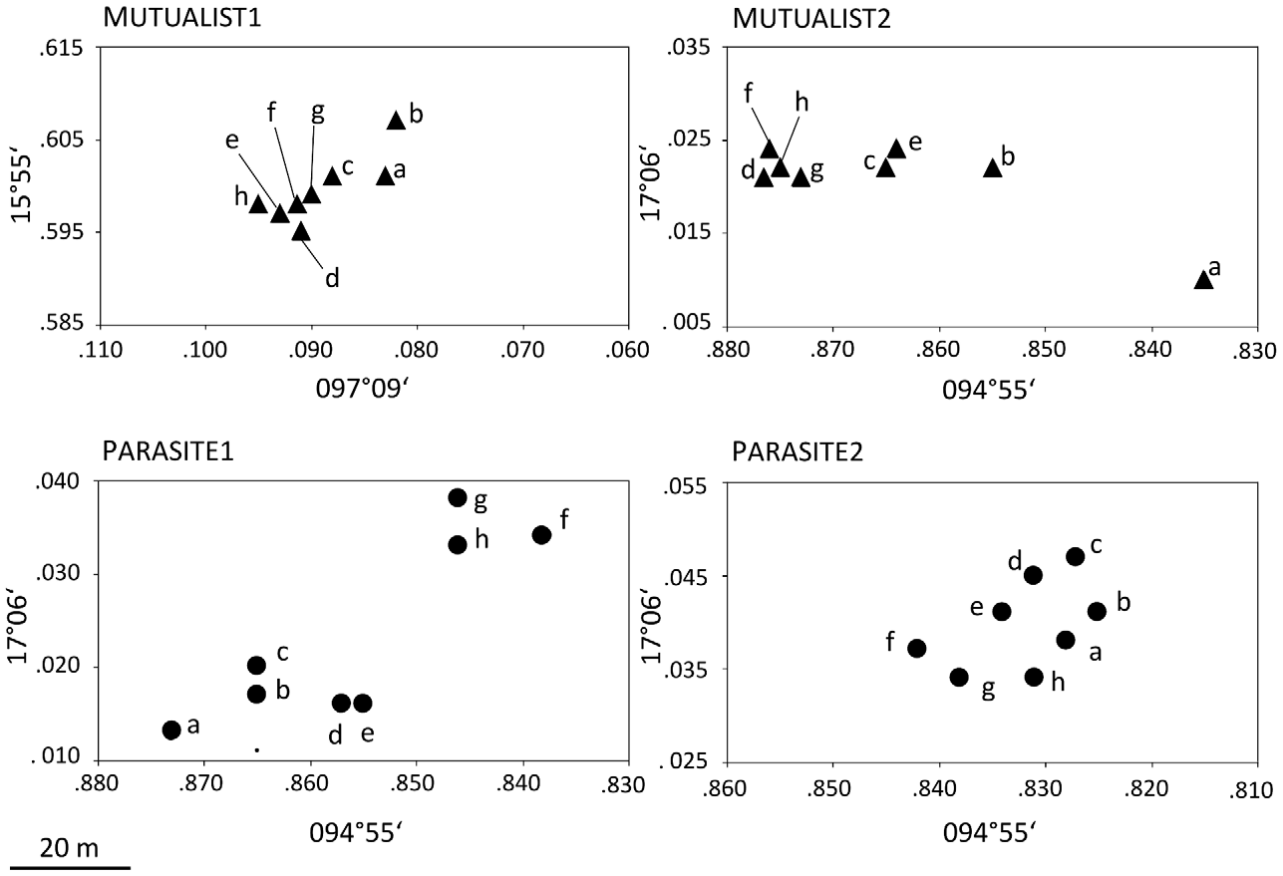
Spatial distribution of the acacia plants from which ants were sampled in each plot. Figures are based on GPS data.

Field studies were conducted in Oaxaca, South Mexico from August to October 2007. The experiments were carried out at two study sites about 150 km apart, one near Puerto Escondido (Pacific coast; ∼15°55′N and ∼097°09′W) and the other one near Matias Romero (Isthmus of Tehuantepec; ∼17°06′N and ∼94°55′W). At the Pacific coastal site, we included ant colonies that inhabited *Acacia hindsii* while we used colonies residing on *Acacia chiapensis* in the Isthmus of Tehuantepec. Plant and ant species were identified following Janzen [26] and Ward [27], respectively. Both acacia species are myrmecophytes that provide hollow swollen thorns as nesting space and food rewards in form of food bodies and extrafloral nectar. For each ant species, two plots with eight trees each were investigated. In each plot, we only included plants from one acacia species. Plots of the mutualist *P. ferrugineus* were termed Mutualist1 (near Puerto Escondido - all *A. hindsii*) and Mutualist2 (near Matias Romero - all *A. chiapensis*), while the plots of the parasite *P. gracilis* were Parasite1 and Parasite2 (both near Matias Romero - all *A. chiapensis*). The eight individual acacias were designated a to h in each plot. We selected the eight closest trees that were inhabited by the same ant species. Foliage of selected trees was not in contact with neighboring acacias. GPS data for each tree trunk were recorded (Fig. 1; Table S1) and pairwise distances were calculated with the Coordinate Distance Calculator (http://boulter.com/gps/distance; Jan-14-2009). Individual ants were given consecutive numbers for all individuals derived from the same host tree. For each tree we sampled ten ant individuals for chemical, six for genetic and five for behavioral analyses.

### Behavioral Trials

In field studies, we tested colony boundaries at the behavioral level within each plot using individual ants. Before transfer, all ants collected from one acacia plant were kept together in a 250 ml plastic cup sealed with fabric (anti-aphid net). Forty workers were transferred from each host tree as follows: five ants were returned to the same tree to test whether ants respond aggressively to an experimentally transferred ant (to serve as control). Another five individuals were placed on each of the other seven trees of the same plot inhabited by the same ant species as the transferred individuals. Ant workers were placed individually onto branches of the study acacias. Behavior was either classified as ‘aggressive’ when the transferred ant was attacked (e.g., mandible opening, chasing, pairwise reciprocal stinging, one or both opponents falling off the tree), or as ‘neutral’ when no attack occurred.

### Cuticular Compounds

Cuticular compounds were sampled from individual ants immediately after collection in the field. Swollen thorns used for the behavioral trials were collected and placed in Ziploc® bags. Ants were killed by freezing at −20°C and then individually placed inside the insert of a GC-vial and washed with 50 µl dichloromethane for 10 minutes. To obtain cuticular profiles with distinct mass spectra, we additionally pooled ten individuals for each ant species in one extract using 200 µl dichloromethane. Each extract was transferred into glass capillaries (disposable micropipettes with ring mark; Blaubrand® intraMARK, Buddeberg GmbH, Mannheim, Germany), and samples were transported to the laboratory for analysis. We aimed to collect cuticular hydrocarbons from ten individuals per acacia but a few samples were lost during transport (22 out of 360; 6.1%).

Identification of cuticular chemicals was conducted using an Agilent 6890 N gas chromatograph coupled to an Agilent 5973 inert mass selective detector. The GC was equipped with an RH-5 ms+capillary column (30 m×0.25 mm i.d.; df = 0.25 µm; J&W Scientific). The program for separation was 70°C initial temperature (1 min), 30°C·min^−1^ to 180°C, then 5°C·min^−1^ to 310°C. Helium was used as the carrier gas with a constant flow rate of 1 ml min^−1^. A split/splitless injector was used (250°C) with the purge valve opened after 60 s. The electron impact mass spectra were recorded with an ionization voltage of 70 eV, a source temperature of 230°C, and an interface temperature of 315°C. We used MSD ChemStation Software for Windows (Agilent Technologies, Palo Alto, CA, USA) for data acquisition. We identified n-alkanes and alkenes by comparing mass spectra with data from a commercial MS library, and methyl and dimethyl alkanes by diagnostic ions and standard MS databases (NIST, Gaithersburg, MD, USA), and by determining Kovats indices following Carlson and co-workers [28]. After identification of peaks based on the mass spectra, we quantified the compounds in each sample using a different GC-MS system (Trace GC Ultra DSQ; Thermo Electron, Austin, TX, USA). The program for separation (SLB™ (5 MS, Supelco, Bellefonte, PA, USA)), 15 m×0.25 mm i.d.; df = 0.25 µm) was used as described above with Helium at a constant flow of 1.5 ml·min^−1^ as carrier gas. The software Xcalibur (Thermo Electron) was used for data acquisition. For the statistical analysis, standardized peak areas were calculated for each individual. We transformed the data to log contrasts to compensate for the non-independence of compositional data. The number of variables was reduced by principal components analysis (PCA) and the data were analyzed by discriminant analysis (DA) using the predefined grouping according to ‘host tree’ using Statistica 6 (statsoft) following Ugelvig and co-workers [29].

### Genetic analyses

We sampled individual ant workers collected from each acacia plant to compare the variation of the cuticular profile and of the behavioral responses with that of neutral genetic markers. DNA extraction and microsatellite analysis was conducted for six workers from each acacia tree (n = 192 workers in total) as described previously [30]. Primer sequences of twelve primer pairs for *P. ferrugineus* were obtained from Kautz and co-workers [31] and of nine primer pairs for *P. gracilis* from Schmid and co-workers [32].

The number of alleles, allele frequencies, expected heterozygosity, and observed heterozygosity at each microsatellite locus for each plot were calculated using the online version of the Genepop software [33]. We calculated pairwise genetic differentiation among groups (each group comprising all workers of one individual host) (*F*_ST_) in Arlequin ver 3.11 [34] to describe genetic structure of ants in each plot. The software Convert [35] was used for allelic data conversion to the appropriate software. To estimate both the number of queens (and queen matings) in each plot, we conducted parentage analysis and inferred sibling groups based on maximum likelihood as implemented in Colony version 1.2 [36]. This approach uses group likelihood ratios based on multi-locus genotypes to partition individuals of haplodiploid species into full-sib and half-sib families. Without prior knowledge of the rate of allelic dropouts or other sources of typing errors, we assumed a realistic error rate of 0.01 for all loci [36]–[38]. First, we assumed only full-sib families and allowed no half-sib relationships. This scenario corresponds to singly mated queens (monoandry). Second, we allowed full-sib families to be nested in half-sib families to test for multiply mated queens (polyandry). We estimated pairwise relatedness of workers collected from one acacia tree using the program Kinship 1.1.2 [39].

### Correlations between geography, genetic colony structure, surface chemistry and behavior

A Mantel test is often used to test for a significant association between two distance matrices [40]. Here, Mantel tests were used to test for associations between pairwise geographic, genetic, chemical and behavioral distances within each experimental plot. The workers collected from individual acacias represented the groups in our statistical design. Partial Mantel correlation tests using distance matrices from geographic (in meters), genetic (as pairwise *F*_ST_), chemical (as Mahalanobis distances received from the discriminant analysis), and behavioral distances (as proportion of aggressive interactions) were carried out in Arlequin ver 3.11 [34] using 2000 permutations. Two-tailed *P*-values are reported.

## Results

### Behavioral Trials

**Figure 2:**
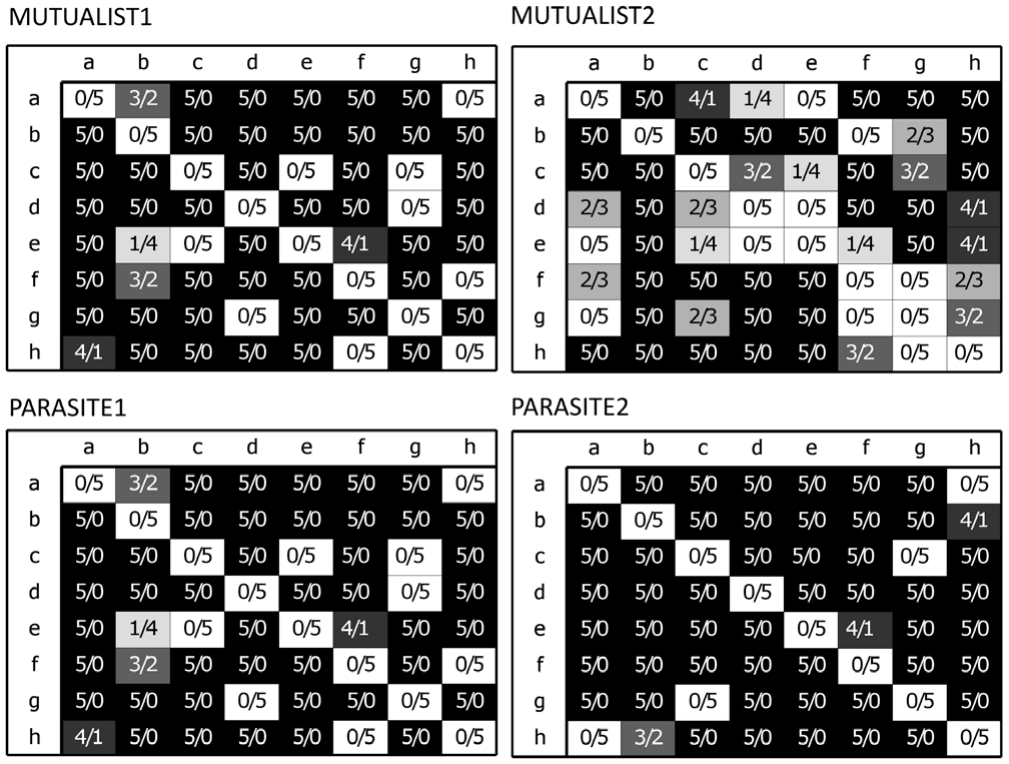
Aggressiveness of ants after replacement within plots. Each plot consisted of eight trees. Five ant individuals from one tree (rows) were individually placed on another tree (columns) and the encounter with an ant individual from the tree it was placed on was observed. Behavior was either classified as aggressive (black background), neutral (white background) or ambiguous (grey shades with darker shades indicating a higher proportion of aggressive encounters). Numbers of ants that reacted aggressive or neutral are indicated in the boxes (aggressive/neutral).

The origin of the transferred ants determined the outcome of the behavioral trials. When ants were collected from one acacia and then placed back onto the same tree, the encounter was neutral in all cases for both species (n = 160 tests with five replicates per pairwise combination; Fig. 2, diagonals). In the ‘between-tree’ tests (for both species total n = 1120 tests; Fig. 2), neutral behavior was detected in 10.7% of the pairwise combinations among all five replicates. In 59.8% of the pairwise combinations, behavior was aggressive in all five trials. In the remaining 29.5% of pairwise combinations, behavior of workers was classified as neutral in some of the five replicates per combination whereas the other encounters were aggressive (grey shading in Fig. 2). In both plots, the mutualist *P. ferrugineus* showed lower levels of aggression towards conspecifics (59% and 72% of all encounters in plots Mutualist1 and Mutualist2, respectively, were aggressive; n = 320 replicates per plot) than the parasite *P. gracilis* (73% and 80% of all encounters in plots Parasite1 and Parasite2 of aggressive nature respectively; n = 320 replicates per plot). Even though we did not further differentiate behaviors within the category ‘aggressive’ in our experiments, we observed differences in the degree of aggressiveness of each species to non-nestmates. In plot Mutualist1, response time of ants was long, sometimes reaching almost 5 minutes. In the other three plots, response time was considerably shorter (usually <30 sec). Aggression between *P. gracilis* intruder and resident individuals seemed more pronounced than in *P. ferrugineus* pairs as encounters escalated rapidly and often resulted in the death of at least one of the ants.

### Cuticular Compounds

**Figure 3:**
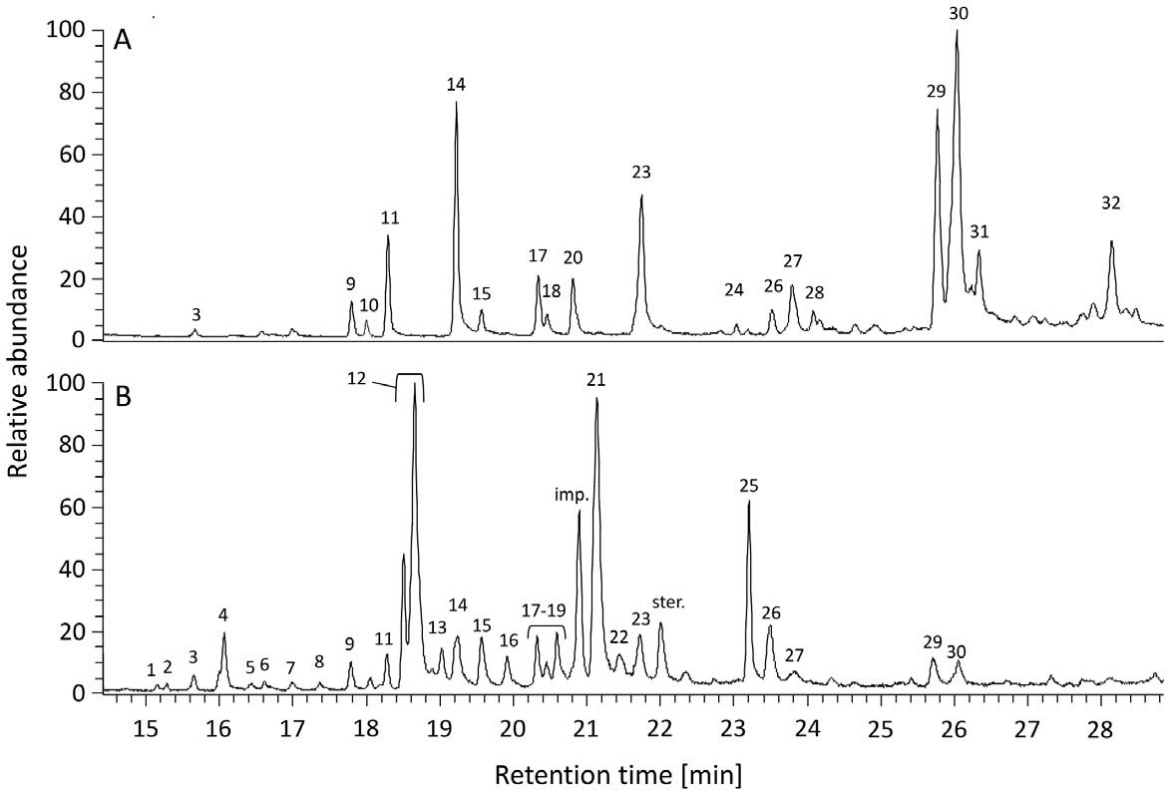
Representative cuticular hydrocarbon profile of the mutualist *Pseudomyrmex ferrugineus* (A) and the parasite *Pseudomyrmex gracilis* (B). The profile of *P. gracilis* consisted of 26 hydrocarbons and the profile of *P. ferrugineus* of 18 hydrocarbons. Peak numbers correspond to the compounds as indicated in Table S2, ‘imp.’ denotes impurity, ‘ster.’ denotes steroid. Pooled extracts from 10 ant individuals.

Gas chromatographic-mass spectrometric (GC-MS) analyses of cuticular compounds identified a total of 18 cuticular compounds for *Pseudomyrmex ferrugineus* and 26 for *P. gracilis* (Fig. 3 and Table S2). We did not consider peaks that had a relative abundance <0.5% in all samples of each species. The compounds varied in chain length between C27 and C37. Although some n-alkanes and n-alkenes were present, the majority of the compounds were mono- and dimethyl-alkanes.

**Figure 4:**
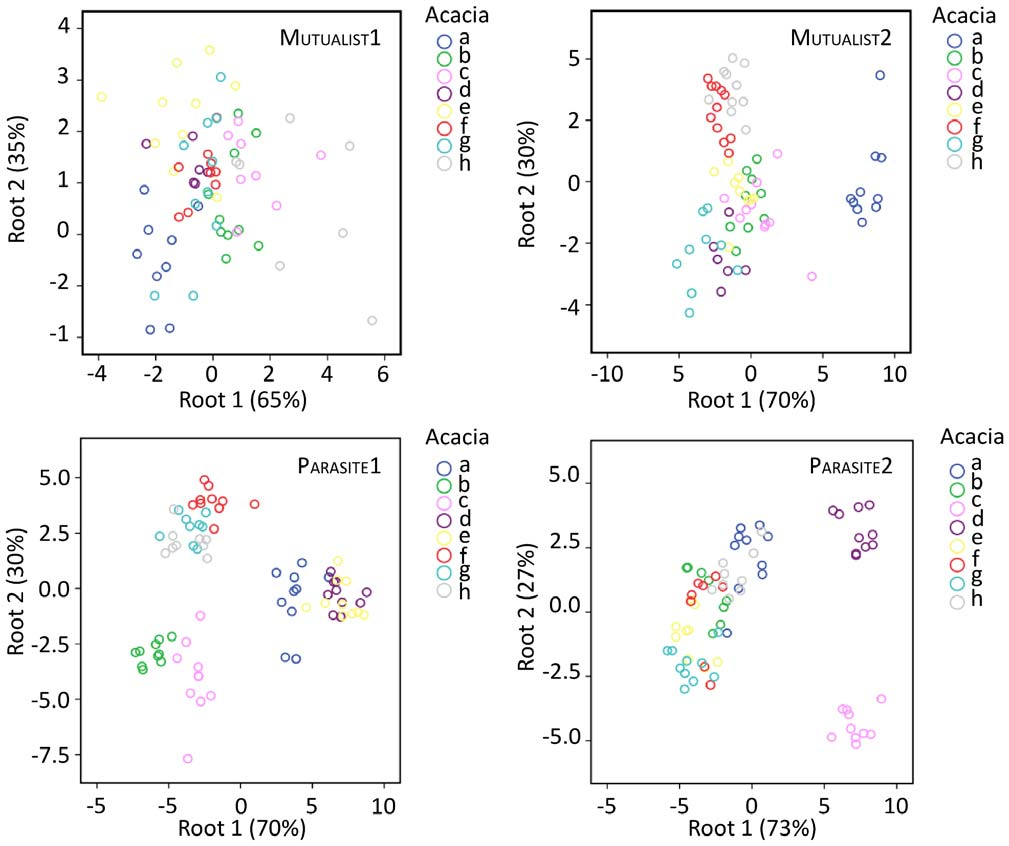
Discriminant analyses of ant hydrocarbon profiles. Hydrocarbon profiles of all individuals that were collected from the eight acacias from each plot are plotted according to their distribution along with the first and second root extracted. Percentages on axes indicate the variance explained by the respective root.

We subjected all 18 hydrocarbons that were regularly observed in the mutualist *P. ferrugineus* to a principal component analysis (PCA). Using eigenvalues greater than one, four principal components were extracted in plot Mutualist1 that together explained 73% of the total variance. Discriminant analysis (DA) based on these four principal components and using ‘host tree’ as a grouping variable showed significant differences of cuticular profiles between the mutualistic ants captured from different acacias (Fig. 4; Wilks' lambda: 0.112; F_28,210_ = 6.28; *P*<0.0001) in plot Mutualist1. In 23 of 28 pairwise comparisons (82%) Mahalanobis distances were significant (Table S3) and 62% of individuals were correctly assigned to their original host tree (n = 69; Table S4). In plot Mutualist2, four principal components extracted in a PCA explained 75% of the variance. According to DA, ants from different individual acacias showed significantly different cuticular chemical profiles (Fig. 4; Wilks' lambda: 0.00395; F_35,263_ = 20.52; *P*<0.0001), whereas all ants collected from the same acacia always clustered closely (indicating highly similar cuticular profiles). All pairwise Mahalanobis distances were significant (Table S3), and 85% of ant individuals were correctly assigned to their group (n = 74; Table S4).

In plot Parasite1, 26 hydrocarbons were subjected to a PCA and six principal components (explaining 83% of the total variance) were extracted. A DA using ‘host tree’ as the grouping variable revealed that the individuals sampled from each acacia always clustered very closely and that individuals taken from the same host plant showed cuticular profiles that were characteristic for that individual acacia tree (Fig. 4; Wilks' lambda: 0.000661; F_42,308_ = 27.63; *P*<0.0001). Mahalanobis distances were significant in 26 of 28 pairwise comparisons (93%), in which all ants from one host tree functioned as a single group (Table S3). Altogether, 87% of individuals were correctly assigned to their original acacia (n = 78; Table S4). In plot Parasite2, five principal components extracted explained 82% of the total variance. Individuals collected from the same tree always clustered very closely and colonies were distinct (Fig. 4; Wilks' lambda: 0.000972; F_35,263_ = 31.59; *P*<0.0001). All pairwise Mahalanobis distances were significant (Table S3). Workers were correctly assigned to their respective colony in 82% of the cases (n = 74; Table S4).

### Genetic diversity and differentiation

All microsatellite loci were polymorphic in every plot. Within each plot, we found 2–11 alleles per locus for *Pseudomyrmex ferrugineus* and 2–19 alleles per locus for *P. gracilis* (Tables 1 and 2). Heterozygosity ranged from 0.07–1.00 (mean 0.67) in *P. ferrugineus* (mutualist) and from 0.29–0.96 (mean 0.73) in *P. gracilis* (parasite). Tests for conformity of genotype proportions to Hardy-Weinberg expectations revealed that most loci showed significant deviation (*p*<0.05, Tables 1 and 2). These deviations can be explained by the fact that workers inhabiting the same acacia are often related and, thus, do not represent independent samples. Pairwise genetic differentiation (*F*_ST_) results were similar in all four plots. 75% to 89% of *F*_ST_ values showed significant differentiation between pairs of groups, i.e. 25 of 28 group pairs were genetically significantly different in plots Mutualist1, Mutualist2 and Parasite1, while *F*_ST_ values of 21 of 28 group pairs were significant in plot Parasite2 (Table S3). Significant group pairwise *F*_ST_ values averaged 0.40±0.08 (mean ± SD, n = 25, range 0.25–0.55) in plot Mutualist1, 0.33±0.05 (n = 25, range 0.26–0.41) in plot Mutualist2, 0.25±0.08 (n = 25, range 0.07–0.39) in plot Parasite1 and 0.19±0.10 (n = 21, range 0.06–0.35) in plot Parasite2.

**Table 1:** Genetic diversity measures within each study plot of the mutualist *Pseudomyrmex ferrugineus* in South Mexico as obtained from female genotypes.

	Mutualist1 (n = 44)			Mutualist2 (n = 47)		
Locus	*N*_A_	*H*_E_	*H*_O_	*N*_A_	*H*_E_	*H*_O_
Psfe14	5	0.68	0.86*	11	0.88	0.96*
Psfe17	6	0.77	0.57*	10	0.87	0.85*
Psfe20	3	0.25	0.07*	5	0.70	0.47*
Psfe21	5	0.69	0.93*	3	0.46	0.62*
Psfe15	4	0.19	0.20	9	0.81	0.64*
Psfe16	6	0.76	0.73*	8	0.86	1.00*
Psfe18	3	0.19	0.21	9	0.87	1.00*
Psfe19	9	0.74	0.66*	6	0.62	0.64*
Psfe06	4	0.65	0.68*	10	0.86	0.83*
Psfe07	3	0.60	0.49*	8	0.84	1.00*
Psfe08	2	0.50	0.52	8	0.84	0.70*
Psfe13	3	0.65	0.61*	11	0.88	1.00*
Total	53			98		
Mean	4.4	0.56	0.54	8.1	0.79	0.81

n denotes the total number of female individuals for each plot; *N*_A_ denotes observed number of alleles found at each locus from each plot; *H*_E_ = expected heterozygosity; *H*_O_ = observed heterozygosity;

* significant deviation according to HW-Probability test (*P*<0.05).

**Table 2:** Genetic diversity measures within each study plot of the parasite *Pseudomyrmex gracilis* in South Mexico as obtained from female genotypes.

	Parasite1 (n = 48)			Parasite 2 (n = 48)		
Locus	*N*_A_	*H*_E_	*H*_O_	*N*_A_	*H*_E_	*H*_O_
Psgr03	8	0.80	0.62*	8	0.82	0.83*
Psgr04	12	0.89	0.94*	11	0.86	0.91
Psgr05	7	0.61	0.54*	5	0.67	0.51*
Psgr06	2	0.43	0.63*	2	0.25	0.29
Psgr07	7	0.81	0.96*	7	0.71	0.80*
Psgr09	4	0.62	0.67*	3	0.66	0.52
Psgr10	13	0.89	0.88*	19	0.91	0.92*
Psgr11	8	0.75	0.83*	9	0.74	0.68
Psgr12	9	0.82	0.85*	13	0.81	0.67*
Total	80			64		
Mean	8.9	0.74	0.77	7.1	0.71	0.68

n denotes the total number of female individuals for each plot; *N*_A_ denotes observed number of alleles found at each locus from each plot; *H*_E_ = expected heterozygosity; *H*_O_ = observed heterozygosity;

* significant deviation according to HW-Probability test (*P*<0.05).

### Genetic colony structure and relatedness

We grouped all sampled individuals from one plot into full-sib (monoandry) and half-sib families using Colony [36]. For the mutualist *P. ferrugineus*, worker genotypes from all but one acacia could be explained by a single queen that had mated once, indicating monogyny and monoandry. The exceptions were acacias 2c and 2e from plot Mutualist2 where the worker genotypes required the assumption of two matings of one queen. Six full-sib families were reconstructed in plot Mutualist1 and six full-sib and one half sib families in plot Mutualist2 indicating that in three of 16 cases a single colony inhabited two host trees. On average, the worker offspring of one *Pseudomyrmex ferrugineus* queen inhabited 1.33 acacias in plot Mutualist1 and 1.6 acacias in plot Mutualist2.

The family structure of the parasite *P. gracilis* revealed 15 full-sib family groups in plot Parasite1. On average, one acacia housed the worker offspring of 1.88 *P. gracilis* queens in plot Parasite1. When assuming multiple mating in this plot, seven half-sib family groups are formed from the 15 full-sib groups. In plot Parasite2, a total of 21 full-sib family groups were formed. Correspondingly, workers inhabiting one tree were offspring of an average of 2.63 *P. gracilis* queens. Under the assumption of multiple matings, each eight half-sib family groups were formed containing a total of 21 full-sib family groups. Based on analysis of twig-nesting populations of this species, single mating seems to dominate in *P. gracilis* (V. Schmid, pers. comm.). Thus, we assume that queens are also more likely to have mated once in our study. Based on our data, we cannot draw conclusions on whether several colonies peacefully shared one acacia or whether *P. gracilis* can be oligogynous. Most importantly, our data clearly demonstrate that a single *P. ferrugineus* queen produces the entire worker force on an acacia whereas several queens do so in *P. gracilis*.

The overall relatedness was estimated among workers derived from one acacia. For *P. ferrugineus* from the plot Mutualist1, relatedness ranged from 0.49±0.31 (mean ± SD) to 0.89±0.08. Results were similar in plot Mutualist2 with average relatedness ranging from 0.44±0.27 to 0.77±0.08 (Table 3). Observed relatedness among workers of *P. gracilis* derived from individual trees in plot Parasite1 varied between 0.11±0.18 and 0.82±0.02, while at plot Parasite2 we found even lower values of relatedness among workers derived from single acacias ranging from 0.00±0.18 to 0.74±0.12 (Table 3). On average, relatedness among workers sampled from the same acacia was 0.71 in the mutualist ant species, compared to 0.42 in the parasitic ant species. Thus, mean relatedness of workers inhabiting the same acacia was 1.69 times higher in the mutualist than in the parasite.

**Table 3:** Relatedness (mean ± SD; R-value) among the workers sampled from each acacia.

Acacia	R-value	n
Mutualist1		
1a	0.89±0.08*	6
1b	0.82±0.09*	6
1c	0.76±0.08	5
1d	0.76±0.07	6
1e	0.82±0.07*	5
1f	0.49±0.31*	5
1g	0.73±0.14	5
1h	0.83±0.06*	6
Mutualist2		
2a	0.77±0.08	6
2b	0.73±0.11	6
2c	0.46±0.25*	6
2d	0.70±0.13	5
2e	0.44±0.27*	6
2f	0.74±0.14	6
2g	0.68±0.08*	6
2h	0.76±0.09	6
Parasite1		
1a	0.82±0.09*	6
1b	0.66±0.14*	6
1c	0.67±0.15*	6
1d	0.67±0.13*	6
1e	0.65±0.13*	6
1f	0.39±0.31*	6
1g	0.28±0.23*	6
1h	0.11±0.18*	6
Parasite2		
2a	0.36±0.16*	6
2b	0.40±0.22*	6
2c	0.62±0.26*	6
2d	0.74±0.12	6
2e	0.12±0.20*	6
2f	0.00±0.18*	6
2g	0.14±0.35*	6
2h	0.11±0.21*	6

Mutualist refers to *Pseudomyrmex ferrugineus*, Parasite to *P. gracilis*.

‘*’ indicates significant deviation from 0.75 (as among full sisters in monogynous colonies) according to T-test. n denotes number of individuals included from each acacia.

### Correlations between geographic, genetic, chemical and behavioral distances

Partial correlation analyses showed that the genetic and chemical distances between the colonies were significantly associated in all four plots (Mantel test; plot Mutualist1: r_gen,chem_ = 0.585, *P* = 0.0015; plot Mutualist2: r_gen,chem_ = 0.384, *P* = 0.0070; plot Parasite1: r_gen,chem_ = 0.613, *P*<0.0001; plot Parasite2: r_gen,chem_ = 0.729, *P* = 0.0250). The geographic and genetic distances between the colonies from each plot were only significantly correlated in plot Mutualist2. The chemical distances between colonies were also only correlated with geographic or behavioral distances in this plot. The behavioral distance between workers derived from one acacia was significantly correlated with geographic, chemical and genetic distance in plots Mutualist2 and Parasite1, but not in plots Mutualist1 and Parasite2. For detailed Mantel test results see Figure S1.

## Discussion

Our behavioral, chemical, and genetic data suggest that the offspring of several queens — likely belonging to different colonies — of the parasite *Pseudomyrmex gracilis* inhabit a single acacia tree. The workers are not aggressive towards each other, possibly because they share the same cuticular hydrocarbon profile from living on the same tree. This is in contrast to the mutualist, *P. ferrugineus*, in which a single colony inhabits one or more acacia trees and ants from different colonies are commonly aggressive towards one another. In *P. ferrugineus*, the genetic distances were concordant with behavioral and chemical distances as all individuals inhabiting the same host plant were full (14 of 16 acacias investigated) or half (two of 16) sisters, showed no aggression amongst each other, and shared similar hydrocarbon profiles.

Ants of both species never showed aggressive behavior after being placed back onto their original host tree (Fig. 2) regardless of their genetic relatedness, indicating that there was no effect of experimental ‘treatment’, i.e., of experimentally removing ants and placing them on their host tree. However, overall aggression among conspecific ants from different trees was high and intruding non-nestmates were usually attacked and chased away or killed by the residents in both species (Fig. 2). *Pseudomyrmex gracilis* workers from different host trees reacted particularly aggressive to one another. Our findings of highly aggressive behavior between ants from spatially separated host trees are in line with a previous study on the same ant species by Clement and co-workers [12]. Therefore, it can be ruled out that the lack of aggressive behavior among ants inhabiting the same tree was caused by a low overall aggressiveness in these species.

Discrimination of non-nestmates occurs after antennal contact and is likely to involve olfactory and tactile perception of cuticular hydrocarbons [20], [21]. For both ant species investigated here, our GC-MS data showed that cuticular hydrocarbon profiles of workers inhabiting the same tree were very similar, which is in accordance with our behavioral observations. Ants obtained from different trees displayed characteristic chemical signatures that were well separated from most (but not all) other groups (Fig. 4), as some *P. ferrugineus* colonies expanded to colonize neighboring host trees.

Despite many exceptions to the rule, monogyny (one queen per colony) and monoandry (single mating per queen) are often viewed as a ‘standard’ in ants [41]. Our genetic analyses showed relatedness values among workers from *P. ferrugineus* colonies around 0.75 and allele counts that indicated monogyny and an effective mating frequency of 1.09 (one out of eleven queens likely mated with two males). High relatedness among workers seems to be common in mutualistic plant-ant systems as described for *Pseudomyrmex peperi*, which is an extremely polygynous acacia mutualist in Central America [30] as well as for the mutualistic *Petalomyrmex phylax*, which inhabits *Leonardoxa africana* in Cameroon [42]. Some *Myrmelachista* species have large polygynous colonies that occupy monospecific patches of understory *Duroia* and *Tococa* ant-plants forming so-called “devil's gardens” [43]. In contrast to the mutualistic plant-ants, relatedness among workers of *P. gracilis* inhabiting a single acacia was often low and sibship reconstruction indicated that the offspring of an average of 2.23 queens shared one host tree. As monogyny and monoandry dominates in other, twig nesting populations of this species (V. Schmid, pers. comm.), it is likely that several unrelated colonies shared a single host tree. Thus, our observations did not necessarily meet our expectations in that non-aggressive behavior was displayed between genetically distinct colonies inhabiting the same tree and sharing similar hydrocarbon profiles.

The apparent contradiction between behavior and hydrocarbon profiles, as well as between hydrocarbons and genetic data, for the parasite *P. gracilis* can most likely be explained by the fact that regardless of the genetic origin, inhabiting the same host plant can lead to similar hydrocarbon profiles [40]. Debout and co-workers [44] observed that workers of the plant-ant *Cataulacus mckeyi*, an exploiter of African *Leonardoxa africana* myrmecophytes, start to rub their antennae on leaves after being experimentally placed onto a different tree. The authors hypothesized that the ants might use this behavior to sequester odors to avoid being attacked by resident ants. We suggest that for the parasite *P. gracilis*, inhabiting the same host tree causes changes to their cuticular signature and consequently their behavior, allowing non-aggressive coexistence of conspecific ants on the same tree regardless of colony structure and genetic differences among individuals. Most likely, this behavior reduces fighting, increases the number of parasitic ants that inhabit a given host and allows for more efficient defense against monopolizing mutualists. Extremely high competition for nesting space has been shown in this system as almost every new thorn produced during the experimental period was occupied by one foundress of co-occurring acacia-ants [18], [30].

In *Pheidole minutula*, cooperative colony founding (pleometrosis) provides a competitive advantage over *Crematogaster laevis*, which compete for the same myrmecophyte *Maieta guianensis* [45]. This has also been reported for other systems [46], [47] and cannot be ruled out for *Pseudomyrmex gracilis*. Pleometrosis is common among ants including territorially-dominant species such as *Oecophylla smaragdina* [48]. Several queens might potentially only be present in the very early stages of colony founding as early workers might admix and later kill all but one queen [6]. Oligogyny, the presence of few queens in mature colonies, is another possible strategy that *P. gracilis* might use to colonize acacia myrmecophytes. Callow workers often lack distinct cuticular hydrocarbons before exhibiting a characteristic gestalt odor, see [49] for an overview. This early ‘cuticular chemical insignificance’ [50] might facilitate admixing of unrelated ants and then lead to a common gestalt among these individuals.

Regardless of the mechanism, the offspring of several unrelated *P. gracilis* queens shared an individual host tree, which is likely to provide an ecological advantage when competing with mutualistic plant-ants as these reach much larger single colony sizes on similar-sized plants than the parasites and thus represent particularly successful competitors of the parasites [12], [18]. Our study further demonstrates that the actual output per *P. gracilis* queen is even lower than previously estimated since not all workers inhabiting the same tree were sisters. Combining results from Clement *et al.* [12] with our own findings, we calculate the median size of *P. ferrugineus* colonies to be 470 workers (upper quartile 705, lower quartile 250) compared to only 36 (upper quartile 46, lower quartile 25) for *P. gracilis*. *Pseudomyrmex ferrugineus* is highly adapted to its lifestyle as an obligate mutualist in terms of social organization. These ants form large and long-lived colonies, invest more energy in colony growth than reproduction, and can thereby defend their host effectively over long periods of time [9], [12], [51].

### Conclusions

Two different colonization strategies used by different acacia-ant species, a mutualist and a parasite of the mutualism, might be driven by interspecific competition and can be explained by the ants' different life histories. The mutualists establish long-term associations with their host plants on which they depend completely. The parasites primarily use the host for nesting space, establish short-lived colonies, and do not completely depend on their host plant. However, large numbers of workers are required to monopolize myrmecophytes as the plants provide numerous hollow swollen thorns for the resident ants and constantly grow producing more nesting space. Consequently, various colonies were found to share a single host in the case of the parasite, *P. gracilis*, and workers inhabiting the same host displayed similar cuticular hydrocarbon profiles. This similarity, which was partly independent of the genetic relatedness of the ants, allowed the non-aggressive coexistence of workers which — according to our microsatellite data — were derived from different queens in most cases. In contrast, the mutualist achieved its stable association with its host by a single colony monopolizing the host, and defending it from conspecific intruders, ultimately reducing the opportunity for other ant colonies to become established. In summary, the specific life history strategy employed by each acacia-ant species shapes the social organization of the resident ants and contrasting strategies may allow the two competing species to coexist in geographic space and evolutionary time.

## Supporting Information

Figure S1 **Partial correlations between genetic, chemical, behavioral and geographic distance.** Host trees were used as grouping variable. Correlation coefficients (r_x,y_) are given for each plot. Mantel tests showed that correlation coefficients were only significant for chemical vs. genetic distance.
(PDF)

Table S1 **GPS Data of collection sites.** GPS data of collection sites are given for each of eight acacia in the plots Mutualist1, Mutualist2, Parasite1, Parasite2.
(PDF)

Table S2 List of compounds identified by GC-MS of dichloromethane extracts of the cuticle of the mutualist *P. ferrugineus* (Mut) and the parasite *P. gracilis* (Par).
(PDF)

Table S3 Colony differentiation based on genetic, chemical, behavioral and geographic pairwise distances.
(PDF)

Table S4 Correct and incorrect posterior assignment of individual workers to their group (using the acacia of which the ant workers were collected as grouping variable) based on cuticular hydrocarbon profiles.
(PDF)
