# Supplementary figures and images for: Host Plant Use by Competing Acacia-Ants: Mutualists Monopolize While Parasites Share Hosts
Source: PLoS One. 2012 May 25;7(5):e37691. doi: 10.1371/journal.pone.0037691 (PMC3360759; doi:10.1371/journal.pone.0037691)

**Figure S1**

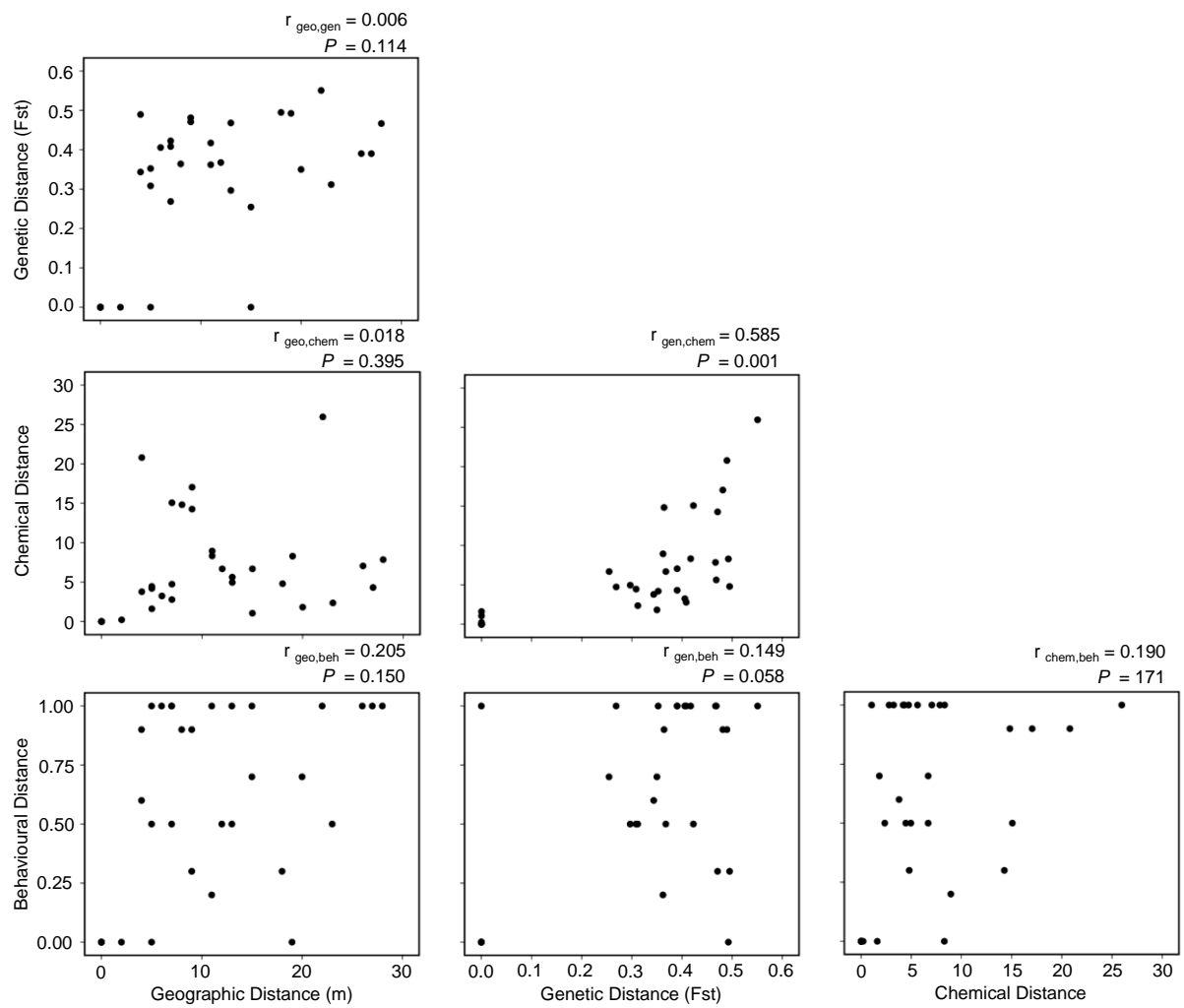

I. Plot MUTUALIST1

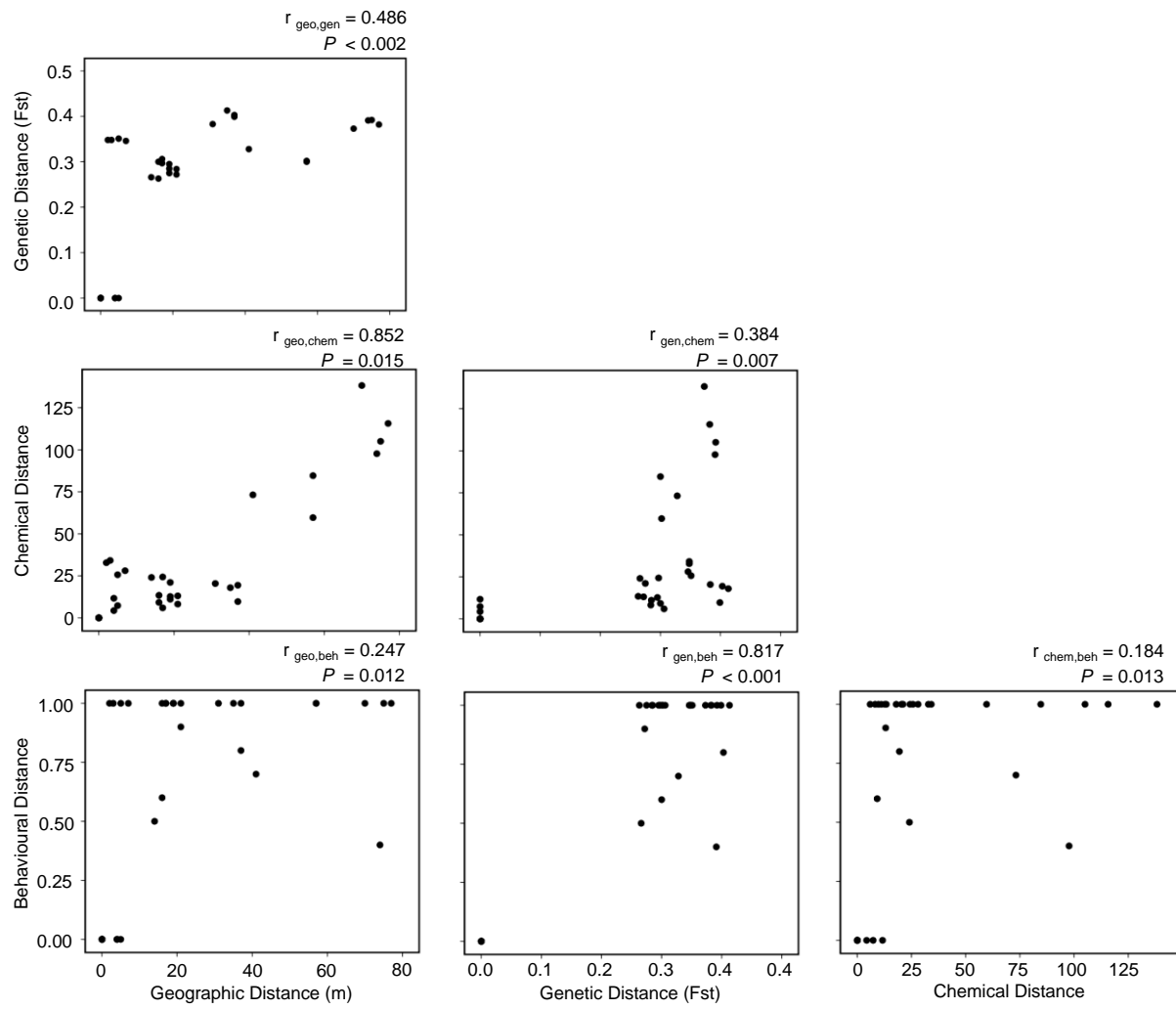

## II. Plot MUTUALIST2

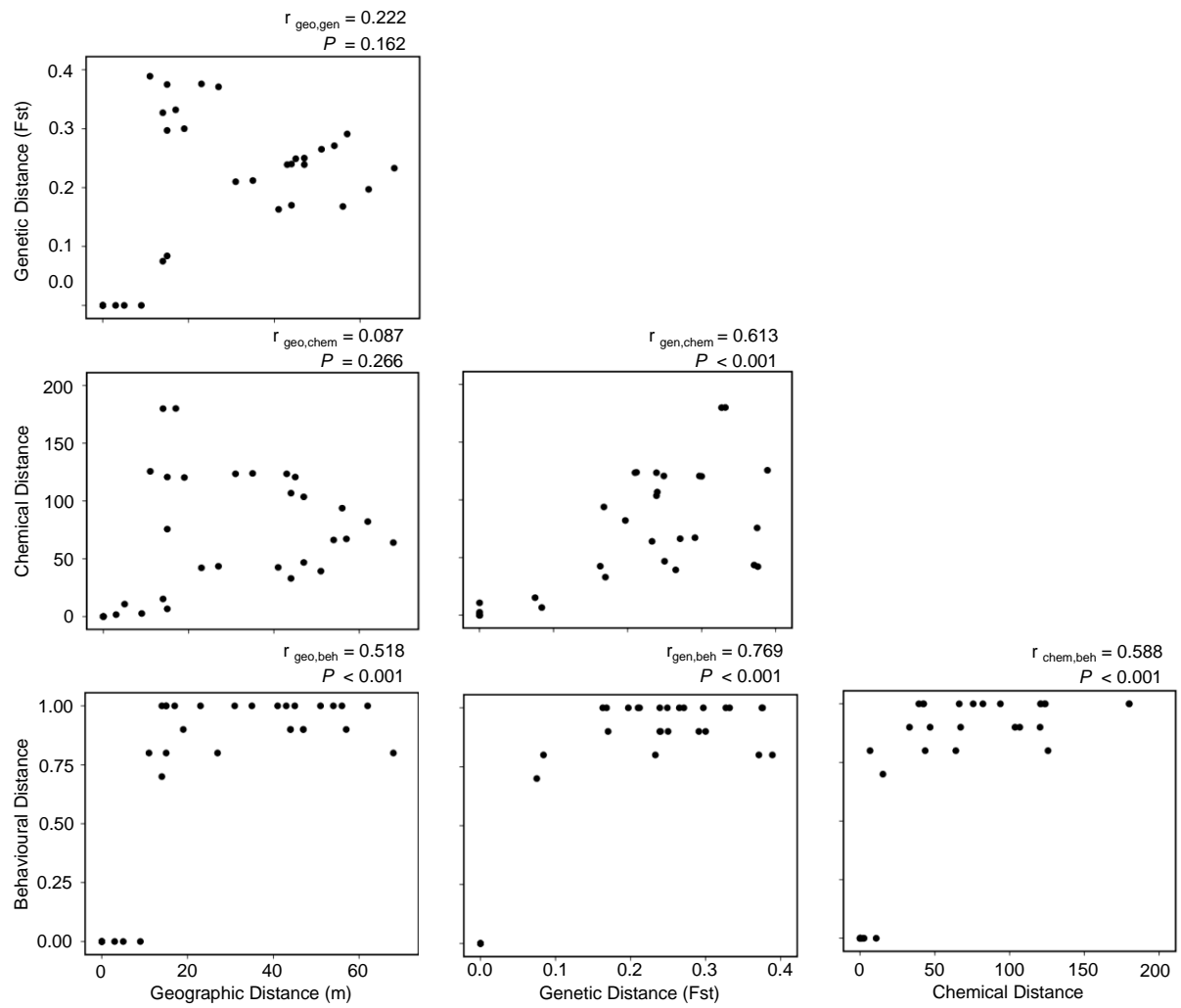

### III. Plot PARASITE1

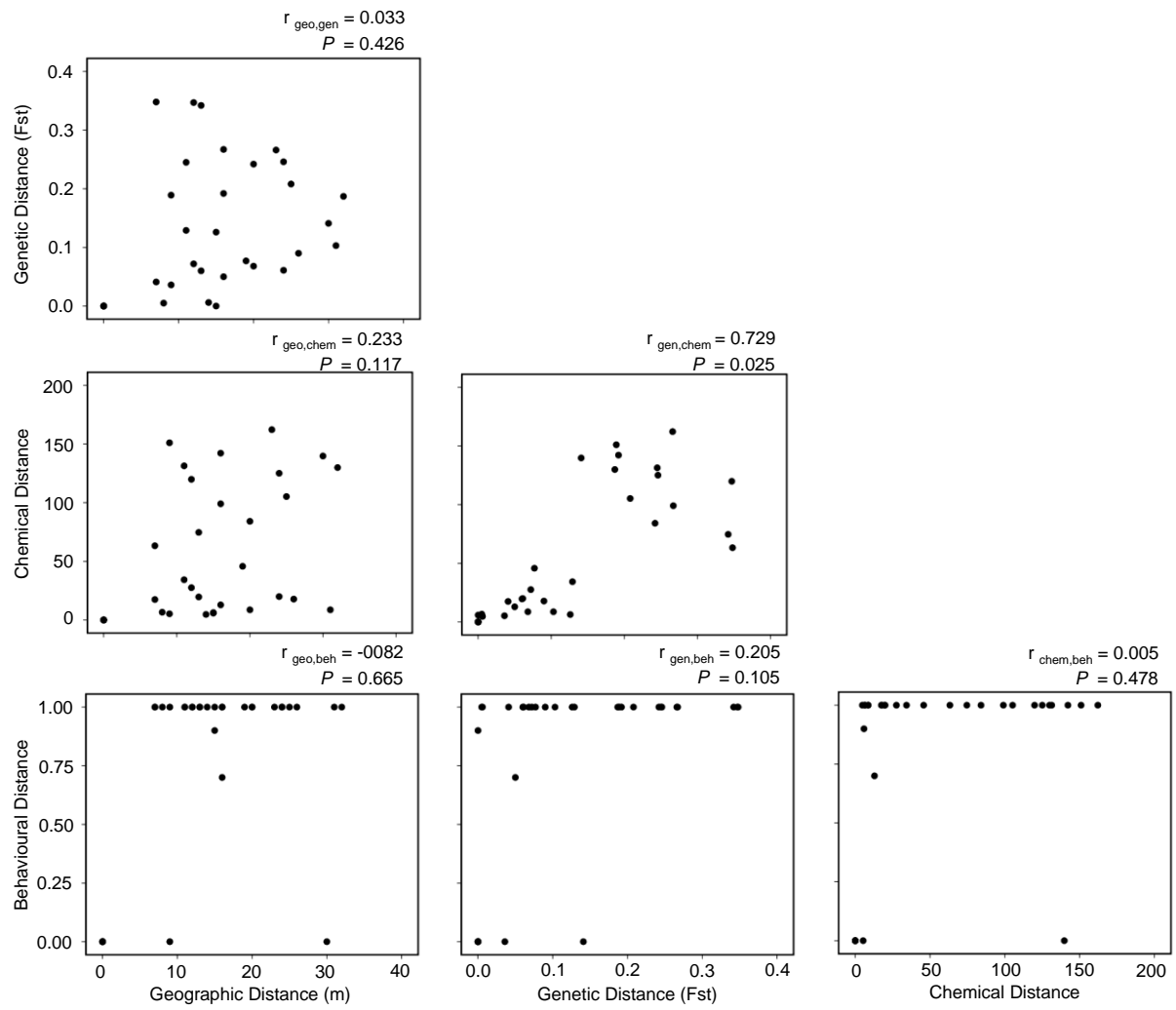

#### IV. Plot PARASITE2

Supplement: Figure S1 — Partial correlations between genetic, chemical, behavioral and geographic distance. Host trees were used as grouping variable. Correlation coefficients (rx,y) are given for each plot. Mantel tests showed that correlation coefficients were only significant for chemical vs. genetic distance. (PDF) [file pone.0037691.s001.pdf]
