# Supplementary material for: Host Plant Use by Competing Acacia-Ants: Mutualists Monopolize While Parasites Share Hosts
Source: PLoS One. 2012 May 25;7(5):e37691. doi: 10.1371/journal.pone.0037691 (PMC3360759; doi:10.1371/journal.pone.0037691)
Supplement: Table S1 — GPS Data of collection sites. GPS data of collection sites are given for each of eight acacia in the plots Mutualist1, Mutualist2, Parasite1, Parasite2. (PDF) [file pone.0037691.s002.pdf]

Table S1

Acacia	GPS N	GPS W	Elevation (ft)
MUTUALIST1			
1a	15°55.601	97°09.083	49
1b	15°55.607	97°09.082	39
1c	15°55.601	97°09.088	44
1d	15°55.595	97°09.091	43
1e	15°55.597	97°09.093	55
1f	15°55.598	97°09.091	55
1g	15°55.599	97°09.090	50
1h	15°55.598	97°09.095	48
MUTUALIST2			
2a	17°06.010	94°55.835	474
2b	17°06.022	94°55.855	438
2c	17°06.022	94°55.865	430
2d	17°06.021	94°55.876	403
2e	17°06.024	94°55.864	447
2f	17°06.024	94°55.876	416
2g	17°06.021	94°55.873	447
2h	17°06.022	94°55.875	429
PARASITE1			
1a	17°06.013	094°55.855	440
1b	17°06.017	094°55.850	462
1c	17°06.020	094°55.850	473
1d	17°06.016	094°55.842	477
1e	17°06.016	094°55.840	465
1f	17°06.034	094°55.823	496
1g	17°06.038	094°55.831	491
1h	17°06.033	094°55.831	506
PARASITE2			
2a	17°06.038	94°55.828	464
2b	17°06.041	94°55.825	467
2c	17°06.047	94°55.827	460
2d	17°06.045	94°55.831	459
2e	17°06.041	94°55.834	460
2f	17°06.037	94°55.842	457
2g	17°06.034	94°55.838	461
2h	17°06.034	94°55.831	465
