# Supplementary material for: Host Plant Use by Competing Acacia-Ants: Mutualists Monopolize While Parasites Share Hosts
Source: PLoS One. 2012 May 25;7(5):e37691. doi: 10.1371/journal.pone.0037691 (PMC3360759; doi:10.1371/journal.pone.0037691)
Supplement: Table S2 — List of compounds identified by GC-MS of dichloromethane extracts of the cuticle of the mutualist P. ferrugineus (Mut) and the parasite P. gracilis (Par). (PDF) [file pone.0037691.s003.pdf]

Table S2

Peak number	Retention time [min]	Full compound name	Short compound name	MUT	PAR
1	15.15	4-methylhexacosane	4meC26		x
2	15.29	Δx-heptacosene	C27:1		x
3	15.63	heptacosane	C27	x	x
4	16.06	13-methylheptacosane	13meC27		x
5	16.41	11,15-dimethylheptacosane	11,15dimeC27		x
6	16.62	3-methylheptacosane	3meC27		x
7	16.98	octacosane	C28		x
8	17.30	14-methyloctacosane	14meC28		x
		13-methyloctacosane	13meC28		x
		12-methyloctacosane	12meC28		x
9	17.77	4-methyloctacosane	4meC28	x	x
		3-methyloctacosane	3meC28	x	x
10	18.07	Δx-nonacosene	C29:1	x	
11	18.27	nonacosane	C29	x	x
12	18.65	15-methylnonacosane	15 meC29		x
		13-methylnonacosane	13 meC29		x
		11-methylnonacosane	11meC29		x
13	19.03	11,15-dimethylnonacosane	11,15dimeC29		x
		13,15-dimethylnonacosane	13,15dimeC29		x
		5-methylnonacosane	5meC29		x
14	19.21	3-methylnonacosane	3meC29	x	x
15	19.55	3, x-dimethylnonacosane	3,xdimeC29		x
		triacontane	C30	x	x
16	19.85	14-methyltriacontane	14meC30		x
		12-methyltriacontane	12meC30		x
17	20.32	4-methyltriacontane	4meC30	x	x
18	20.45	3-methyltriacontane	3meC30	x	x
19	20.58	Δx-hentriacontene	C31:1		x
20	20.81	hentriacontane	C31	x	
21	21.14	15-methylhentriacontane	15meC31		x
		13-methylhentriacontane	13meC31		x
		11-methylhentriacontane	11meC31		x
22	21.44	11,15-dimethylhentriacontane	11,15dimeC31		x
23	21.67	3-methylhentriacontane	3meC31	x	x
24	23.00	x, y-dimethyldotriacontane	x, y-dimeC32	x	

25	23.22	14-methyldotriacontane	14meC32		**x**
		12-methyldotriacontane	12meC32		**x**
26	23.50	15-methyltritriacontane	15meC33	**x**	
		13-methyltritriacontane	13meC33	**x**	**x**
		11-methyltritriacontane	11meC33	**x**	**x**
27	23.79	11,15-dimethyltritriacontane	11,15dimeC33	**x**	**x**
		11,12-dimethyltritriacontane	11,12dimeC33	**x**	
28	24.08	unidentified compound	unID	**x**	
29	25.71	17-methylpentatriacontane	17meC35	**x**	**x**
		15-methylpentatriacontane	15meC35	**x**	**x**
		13-methylpentatriacontane	13meC35	**x**	**x**
		11-methylpentatriacontane	11meC35	**x**	**x**
30	26.03	11,15-dimethylpentatriacontane	11,15dimeC35	**x**	**x**
31	26.33	unidentified compound	unID	**x**	
32	28.15	11,x-dimethylheptatriacontane	11,xdimeC37	**x**	
