# Supplementary material for: Host Plant Use by Competing Acacia-Ants: Mutualists Monopolize While Parasites Share Hosts
Source: PLoS One. 2012 May 25;7(5):e37691. doi: 10.1371/journal.pone.0037691 (PMC3360759; doi:10.1371/journal.pone.0037691)
Supplement: Table S3 — Colony differentiation based on genetic, chemical, behavioral and geographic pairwise distances. (PDF) [file pone.0037691.s004.pdf]

Table S3

I. Colony differentiation based on genetic, chemical, behavioral and geographic pairwise distances in plot MUTUALIST1.

a) Pairwise genetic distances (F_{ST}) of *P. ferrugineus* in plot MUTUALIST1 using sampled workers derived from one acacia as grouping variable. Results are based on twelve microsatellite loci (Table 2). Bold numbers indicate significance after multiple comparisons, $P < 0.05$.

	1a	1b	1c	1d	1e	1f	1g	1h
1a	—							
1b	**0.417**	—						
1c	**0.364**	0.000	—					
1d	**0.495**	**0.390**	**0.368**	—				
1e	**0.493**	**0.390**	**0.362**	0.000	—			
1f	**0.255**	**0.312**	**0.269**	**0.352**	**0.344**	—		
1g	**0.297**	**0.350**	**0.308**	**0.408**	**0.406**	0.000	—	
1h	**0.551**	**0.467**	**0.468**	**0.481**	**0.490**	**0.423**	**0.471**	—

b) Pairwise chemical (Mahalanobis) distances based on discriminant analysis of the cuticular hydrocarbon profiles of *P. ferrugineus* in plot MUTUALIST1. Workers derived from one acacia were used as grouping variable (Fig. 4). Bold numbers indicate significance.

	1a	1b	1c	1d	1e	1f	1g	1h
1a	—							
1b	**8.32**	—						
1c	**14.82**	1.05	—					
1d	**4.80**	**4.31**	**6.68**	—				
1e	**8.29**	**7.05**	**8.94**	1.62	—			
1f	**6.69**	**2.36**	**4.73**	**4.19**	**3.78**	—		
1g	**4.96**	1.82	**4.46**	2.79	**3.24**	0.21	—	
1h	**25.97**	**7.85**	**5.63**	**17.04**	**20.80**	**15.07**	**14.27**	—

c) Pairwise behavioral distance (proportions of aggressive encounters) of *P. ferrugineus* in plot MUTUALIST1. Workers derived from one acacia were used as grouping variable (Fig. 2).

	1a	1b	1c	1d	1e	1f	1g	1h
1a	—							
1b	1.0	—						
1c	0.9	1.0	—					
1d	0.3	1.0	0.5	—				
1e	0.0	1.0	0.2	0.0	—			
1f	0.7	0.5	1.0	1.0	0.6	—		
1g	0.5	0.7	0.5	1.0	1.0	0.0	—	
1h	1.0	1.0	1.0	0.9	0.9	0.5	0.3	—

d) Pairwise geographic distances (in m) between the study acacias of *P. ferrugineus* in plot MUTUALIST1 (Fig. 1).

	1a	1b	1c	1d	1e	1f	1g	1h
1a	—							
1b	11	—						
1c	8	15	—					
1d	18	27	12	—				
1e	19	26	11	5	—			
1f	15	23	7	5	4	—		
1g	13	20	5	7	6	2	—	
1h	22	28	13	9	4	7	9	—

II. Colony differentiation based on genetic, chemical, behavioral and geographic pairwise distances in plot MUTUALIST2.

a) Pairwise genetic distances (F_{ST}) of *P. ferrugineus* in plot MUTUALIST2 using sampled workers derived from one acacia as grouping variable. Results are based on twelve microsatellite loci (Table 2). Bold numbers indicate significance after multiple comparisons, $P < 0.05$.

2a	—							
2b	**0.328**	—						
2c	**0.302**	**0.306**	—					
2d	**0.392**	**0.399**	**0.285**	—				
2e	**0.300**	**0.300**	0.000	**0.284**	—			
2f	**0.382**	**0.403**	**0.275**	**0.351**	**0.272**	—		
2g	**0.373**	**0.383**	**0.266**	0.000	**0.263**	**0.346**	—	
2h	**0.391**	**0.413**	**0.297**	**0.348**	**0.295**	0.000	**0.348**	—
	2a	2b	2c	2d	2e	2f	2g	2h

b) Pairwise chemical (Mahalanobis) distances based on discriminant analysis of the cuticular hydrocarbon profiles of *P. ferrugineus* in plot MUTUALIST2. Workers derived from one acacia were used as grouping variable (Fig. 4). Bold numbers indicate significance.

2a	—							
2b	**73.17**	—						
2c	**59.64**	**5.94**	—					
2d	**105.04**	**9.67**	**11.06**	—				
2e	**84.63**	**9.17**	**11.65**	**8.19**	—			
2f	**115.69**	**19.43**	**21.05**	**25.65**	**13.07**	—		
2g	**138.23**	**20.40**	**24.03**	**7.29**	**13.38**	**28.04**	—	
2h	**97.68**	**17.93**	**24.32**	**32.79**	**12.71**	**4.33**	**34.12**	—
	2a	2b	2c	2d	2e	2f	2g	2h

c) Pairwise behavioral distance (proportions of aggressive encounters) of *P. ferrugineus* in plot MUTUALIST2. Workers derived from one acacia were used as grouping variable (Fig. 2).

2a	—							
2b	0.70	—						
2c	1.00	1.00	—					
2d	1.00	1.00	1.00	—				
2e	1.00	0.60	0.00	1.00	—			
2f	1.00	0.80	1.00	1.00	0.90	—		
2g	1.00	1.00	0.50	0.00	1.00	1.00	—	
2h	0.40	1.00	1.00	1.00	1.00	0.00	1.00	—
	2a	2b	2c	2d	2e	2f	2g	2h

d) Pairwise geographic distances (in m) between the study acacias of *P. ferrugineus* in plot MUTUALIST2 (Fig. 1).

2a	—							
2b	41	—						
2c	57	17	—					
2d	75	37	19	—				
2e	57	16	4	21	—			
2f	77	37	19	5	21	—		
2g	70	31	14	5	16	7	—	
2h	74	35	17	2	19	4	3	—
	2a	2b	2c	2d	2e	2f	2g	2h

III. Colony differentiation based on genetic, chemical, behavioral and geographic pairwise distances in plot PARASITE1.

a) Pairwise genetic distances (F_{ST}) of *P. gracilis* in plot PARASITE1 using sampled workers derived from one acacia as grouping variable. Results are based on nine microsatellite loci (Table 3). Bold numbers indicate significance after multiple comparisons, $P < 0.05$.

1a	—							
1b	**0.389**	—						
1c	**0.375**	0.000	—					
1d	**0.376**	**0.327**	**0.297**	—				
1e	**0.371**	**0.332**	**0.300**	0.000	—			
1f	**0.233**	**0.291**	**0.271**	**0.239**	**0.240**	—		
1g	**0.197**	**0.265**	**0.250**	**0.249**	**0.239**	**0.084**	—	
1h	**0.168**	**0.170**	**0.163**	**0.210**	**0.212**	**0.075**	0.000	—
	1a	1b	1c	1d	1e	1f	1g	1h

b) Pairwise chemical (Mahalanobis) distances based on discriminant analysis of the cuticular hydrocarbon profiles of *P. gracilis* in plot PARASITE1. Workers derived from one acacia were used as grouping variable (Fig. 4). Bold numbers indicate significance.

1a	—							
1b	**125.65**	—						
1c	**75.66**	**10.72**	—					
1d	**42.14**	**179.93**	**120.71**	—				
1e	**43.50**	**180.06**	**120.35**	1.66	—			
1f	**64.01**	**67.18**	**66.29**	**103.59**	**106.78**	—		
1g	**82.14**	**39.29**	**46.80**	**120.72**	**123.51**	6.64	—	
1h	**93.83**	**32.99**	**42.52**	**123.53**	**123.87**	**15.22**	**2.61**	—
	1a	1b	1c	1d	1e	1f	1g	1h

c) Pairwise behavioral distance (proportions of aggressive encounters) of *P. gracilis* in plot PARASITE1. Workers derived from one acacia were used as grouping variable (Fig. 2).

1a	—							
1b	0.8	—						
1c	1.0	0.0	—					
1d	1.0	1.0	1.0	—				
1e	0.8	1.0	0.9	0.0	—			
1f	0.8	0.9	1.0	0.9	0.9	—		
1g	1.0	1.0	0.9	1.0	1.0	0.8	—	
1h	1.0	0.9	1.0	1.0	1.0	0.7	0.0	—
	1a	1b	1c	1d	1e	1f	1g	1h

d) Pairwise geographic distances (in m) between the study acacias of *P. gracilis* in plot PARASITE1 (Fig. 1).

1a	—							
1b	11	—						
1c	15	5	—					
1d	23	14	15	—				
1e	27	17	19	3	—			
1f	68	57	54	47	44	—		
1g	62	51	47	45	43	15	—	
1h	56	44	41	31	35	14	9	—
	1a	1b	1c	1d	1e	1f	1g	1h

IV. Colony differentiation based on genetic, chemical, behavioral and geographic pairwise distances in plot PARASITE2.

a) Pairwise genetic distances (F_{ST}) of *P. gracilis* in plot PARASITE2 using sampled workers derived from one acacia as grouping variable. Results are based on nine microsatellite loci (Table 3). Bold numbers indicate significance after multiple comparisons, $P < 0.05$.

2a	—							
2b	0.041	—						
2c	**0.267**	**0.245**	—					
2d	**0.342**	**0.347**	**0.348**	—				
2e	**0.129**	**0.126**	**0.192**	**0.189**	—			
2f	**0.061**	**0.103**	**0.187**	**0.246**	0.000	—		
2g	**0.077**	**0.090**	**0.141**	**0.266**	0.006	0.000	—	
2h	0.036	0.050	**0.208**	**0.242**	**0.060**	**0.068**	0.072	—
	2a	2b	2c	2d	2e	2f	2g	2h

b) Pairwise chemical (Mahalanobis) distances based on discriminant analysis of the cuticular hydrocarbon profiles of *P. gracilis* in plot PARASITE2. Workers derived from one acacia were used as grouping variable (Fig. 4). Bold numbers indicate significance.

2a	—							
2b	**17.46**	—						
2c	**99.04**	**131.38**	—					
2d	**74.64**	**119.89**	**63.27**	—				
2e	**34.27**	**6.32**	**142.19**	**151.00**	—			
2f	**19.95**	**8.63**	**129.94**	**125.07**	**5.83**	—		
2g	**45.76**	**17.78**	**139.79**	**162.21**	**4.75**	**6.61**	—	
2h	**5.28**	**12.87**	**105.23**	**84.09**	**19.60**	**8.66**	**27.52**	—
	2a	2b	2c	2d	2e	2f	2g	2h

c) Pairwise behavioral distance (proportions of aggressive encounters) of *P. gracilis* in plot PARASITE2. Workers derived from one acacia were used as grouping variable (Fig. 2).

2a	—							
2b	1.0	—						
2c	1.0	1.0	—					
2d	1.0	1.0	1.0	—				
2e	1.0	1.0	1.0	1.0	—			
2f	1.0	1.0	1.0	1.0	0.9	—		
2g	1.0	1.0	0.0	1.0	1.0	1.0	—	
2h	0.0	0.7	1.0	1.0	1.0	1.0	1.0	—
	2a	2b	2c	2d	2e	2f	2g	2h

d) Pairwise geographic distances (in m) between the study acacias of *P. gracilis* in plot PARASITE2 (Fig. 1).

2a	—							
2b	7	—						
2c	16	11	—					
2d	13	12	7	—				
2e	11	15	16	9	—			
2f	24	31	32	24	15	—		
2g	19	26	30	23	14	8	—	
2h	9	16	25	20	13	20	12	—
	2a	2b	2c	2d	2e	2f	2g	2h
