# Supplementary material for: Host Plant Use by Competing Acacia-Ants: Mutualists Monopolize While Parasites Share Hosts
Source: PLoS One. 2012 May 25;7(5):e37691. doi: 10.1371/journal.pone.0037691 (PMC3360759; doi:10.1371/journal.pone.0037691)
Supplement: Table S4 — Correct and incorrect posterior assignment of individual workers to their group (using the acacia of which the ant workers were collected as grouping variable) based on cuticular hydrocarbon profiles. (PDF) [file pone.0037691.s005.pdf]

Table S4

Correct and incorrect posterior assignment of individual workers to their group (using the acacia of which the ant workers were collected as grouping variable) based on cuticular hydrocarbon profiles in each plot.

I) Plot MUUALIST1: Posterior probabilities of each individual worker to be assigned to its original acacia in which it was sampled based on the chemical hydrocarbon profile. Cases, in which posterior probabilities were higher for another acacia (bold), this posterior probability, as well as the identification for the other acacia are given.

		Posterior probability for		
Original acacia	Individual	Original acacia	Alternative acacia	Alternative acacia
1a	1a-01	0.83		
1a	1a-02	**0.31**	**0.34**	**1g**
1a	1a-03	**0.02**	**0.47**	**1f**
1a	1a-04	0.88		
1a	1a-05	0.77		
1a	1a-06	0.88		
1a	1a-08	0.97		
1a	1a-09	0.96		
1a	1a-10	0.45		
1b	1b-01	0.48		
1b	1b-02	0.39		
1b	1b-03	**0.26**	**0.58**	**1c**
1b	1b-04	0.53		
1b	1b-05	**0.20**	**0.31**	**1c**
1b	1b-06	0.56		
1b	1b-07	0.30		
1b	1b-08	**0.25**	**0.26**	**1g**
1b	1b-09	0.54		
1b	1b-10	0.36		
1c	1c-01	0.48		
1c	1c-02	0.90		
1c	1c-03	**0.20**	**0.23**	**1b**
1c	1c-04	0.53		
1c	1c-05	**0.35**	**0.41**	**1b**
1c	1c-07	0.51		
1c	1c-08	0.30		
1c	1c-09	**0.12**	**0.43**	**1b**

1c	1c-10	**0.18**	**0.40**	**1f**
1d	1d-01	0.48		
1d	1d-03	**0.15**	**0.28**	**1g**
1d	1d-04	0.24		
1d	1d-08	0.42		
1d	1d-09	0.50		
1d	1d-10	**0.14**	**0.32**	**1g**
1e	1e-01	0.85		
1e	1e-02	0.47		
1e	1e-03	0.56		
1e	1e-04	0.56		
1e	1e-05	0.30		
1e	1e-06	0.82		
1e	1e-07	0.75		
1e	1e-08	**0.26**	**0.30**	**1c**
1e	1e-09	**0.03**	**0.30**	**1g**
1e	1e-10	0.75		
1f	1f-01	0.49		
1f	1f-02	0.49		
1f	1f-03	0.39		
1f	1f-04	**0.04**	**0.44**	**1d**
1f	1f-05	0.44		
1f	1f-06	0.39		
1f	1f-08	**0.30**	**0.33**	**1g**
1f	1f-09	**0.24**	**0.25**	**1e**
1f	1f-10	0.37		
1g	1g-01	**0.05**	**0.91**	**1a**
1g	1g-02	0.40		
1g	1g-03	0.34		
1g	1g-04	**0.33**	**0.55**	**1f**
1g	1g-05	**0.23**	**0.26**	**1e**
1g	1g-07	**0.19**	**0.42**	**1e**
1g	1g-08	**0.13**	**0.49**	**1e**
1g	1g-09	**0.26**	**0.42**	**1a**
1g	1g-10	0.31		
1h	1h-02	0.72		
1h	1h-03	**0.01**	**0.35**	**1b**
1h	1h-04	1.00		

1h	1h-07	**0.23**	**0.41**	**1b**
1h	1h-08	**0.01**	**0.31**	**1b**
1h	1h-09	1.00		

II) Plot MUTUALIST2: Posterior probabilities of each individual worker to be assigned to its original acacia in which it was sampled based on the chemical hydrocarbon profile. Cases, in which posterior probabilities were higher for another acacia (bold), this posterior probability, as well as the identification for the other acacia are given.

		Posterior probability for		
Original acacia	Individual	Original acacia	Alternative acacia	Alternative acacia
2a	2a-01	1.00		
2a	2a-02	1.00		
2a	2a-03	1.00		
2a	2a-04	1.00		
2a	2a-05	1.00		
2a	2a-06	1.00		
2a	2a-07	1.00		
2a	2a-08	1.00		
2a	2a-09	1.00		
2a	2a-10	1.00		
2b	2b-01	0.99		
2b	2b-02	0.91		
2b	2b-03	0.62		
2b	2b-04	**0.46**	**0.54**	**2c**
2b	2b-05	**0.21**	**0.63**	**2c**
2b	2b-06	0.92		
2b	2b-07	0.94		
2b	2b-08	**0.46**	**0.51**	**2e**
2b	2b-09	0.86		
2c	2c-01	0.57		
2c	2c-02	0.56		
2c	2c-03	**0.27**	**0.50**	**2b**
2c	2c-04	0.97		
2c	2c-05	0.98		
2c	2c-06	0.89		
2c	2c-07	0.98		
2c	2c-08	0.99		
2c	2c-09	0.96		
2c	2c-10	0.90		
2d	2d-01	0.97		
2d	2d-04	0.75		

2d	2d-05	0.93		
2d	2d-06	**0.10**	**0.57**	**2c**
2d	2d-08	0.59		
2d	2d-09	0.43		
2e	2e-01	0.48		
2e	2e-02	**0.14**	**0.74**	**2f**
2e	2e-03	0.99		
2e	2e-04	1.00		
2e	2e-05	0.99		
2e	2e-06	0.98		
2e	2e-07	0.53		
2e	2e-08	0.99		
2e	2e-09	**0.22**	**0.61**	**2b**
2e	2e-10	0.93		
2f	2f-01	0.71		
2f	2f-02	0.77		
2f	2f-03	0.86		
2f	2f-04	0.95		
2f	2f-05	0.81		
2f	2f-06	0.82		
2f	2f-07	0.84		
2f	2f-08	0.77		
2f	2f-09	0.91		
2f	2f-10	0.96		
2g	2g-01	1.00		
2g	2g-02	0.97		
2g	2g-03	1.00		
2g	2g-04	1.00		
2g	2g-06	**0.23**	**0.64**	**2d**
2g	2g-07	**0.33**	**0.39**	**2e**
2g	2g-08	**0.20**	**0.79**	**2d**
2g	2g-09	0.48		
2g	2g-10	0.72		
2h	2h-01	0.89		
2h	2h-02	0.95		
2h	2h-03	0.89		
2h	2h-04	**0.48**	**0.51**	**2f**
2h	2h-05	0.99		

2h	2h-07	0.50
2h	2h-08	0.85
2h	2h-09	0.77
2h	2h-10	0.94

III) Plot PARASITE1:Posterior probabilities of each individual worker to be assigned to its original acacia in which it was sampled based on the chemical hydrocarbon profile. Cases, in which posterior probabilities were higher for another acacia (bold), this posterior probability, as well as the identification for the other acacia are given.

		Posterior probability for		
Original acacia	Individual	Original acacia	Alternative acacia	Alternative acacia
1a	1a-01	1.00		
1a	1a-02	1.00		
1a	1a-03	1.00		
1a	1a-04	1.00		
1a	1a-05	1.00		
1a	1a-06	1.00		
1a	1a-07	1.00		
1a	1a-08	1.00		
1a	1a-09	1.00		
1a	1a-10	1.00		
1b	1b-01	0.97		
1b	1b-02	1.00		
1b	1b-03	0.96		
1b	1b-04	1.00		
1b	1b-05	1.00		
1b	1b-06	0.99		
1b	1b-07	0.98		
1b	1b-08	0.99		
1b	1b-09	1.00		
1b	1b-10	0.84		
1c	1c-01	1.00		
1c	1c-02	1.00		
1c	1c-03	1.00		
1c	1c-04	1.00		
1c	1c-05	0.65		
1c	1c-06	0.59		
1c	1c-07	1.00		
1c	1c-08	1.00		
1c	1c-09	1.00		
1c	1c-10	0.70		
1d	1d-01	1.00		

1d	1d-02	0.73		
1d	1d-03	**0.43**	**0.57**	**1e**
1d	1d-04	**0.48**	**0.52**	**1e**
1d	1d-05	0.89		
1d	1d-06	0.66		
1d	1d-07	0.58		
1d	1d-08	**0.28**	**0.72**	**1e**
1d	1d-09	0.51		
1d	1d-10	0.18	0.82	1e
1e	1e-01	0.76		
1e	1e-02	**0.41**	**0.59**	**1d**
1e	1e-03	0.55		
1e	1e-04	0.78		
1e	1e-05	0.61		
1e	1e-06	0.81		
1e	1e-07	0.79		
1e	1e-08	0.81		
1e	1e-09	0.65		
1e	1e-10	0.68		
1f	1f-01	1.00		
1f	1f-02	0.78		
1f	1f-03	0.98		
1f	1f-04	0.99		
1f	1f-05	0.77		
1f	1f-06	0.97		
1f	1f-07	0.81		
1f	1f-08	0.97		
1f	1f-09	**0.25**	**0.52**	**1g**
1f	1f-10	1.00		
1g	1g-01	0.66		
1g	1g-02	0.76		
1g	1g-03	0.76		
1g	1g-04	0.86		
1g	1g-05	0.85		
1g	1g-07	0.62		
1g	1g-08	**0.44**	**0.56**	**1h**
1g	1g-09	0.67		
1g	1g-10	0.80		

1h	1h-01	**0.42**	**0.57**	**1g**
1h	1h-02	**0.45**	**0.54**	**1g**
1h	1h-03	**0.28**	**0.72**	**1g**
1h	1h-04	1.00		
1h	1h-05	0.67		
1h	1h-07	0.86		
1h	1h-08	0.90		
1h	1h-09	0.78		
1h	1h-10	0.71		

IV) Plot PARASITE2: Posterior probabilities of each individual worker to be assigned to its original acacia in which it was sampled based on the chemical hydrocarbon profile. Cases, in which posterior probabilities were higher for another acacia (bold), this posterior probability, as well as the identification for the other acacia are given.

		Posterior probability for		
Original acacia	Individual	Original acacia	Alternative acacia	Alternative acacia
2a	2a-01	0.50		
2a	2a-02	**0.00**	**0.88**	**2b**
2a	2a-03	0.99		
2a	2a-04	1.00		
2a	2a-05	0.94		
2a	2a-06	0.96		
2a	2a-07	0.98		
2a	2a-08	**0.03**	**0.88**	**2b**
2a	2a-09	1.00		
2a	2a-10	0.98		
2b	2b-03	0.94		
2b	2b-04	0.62		
2b	2b-05	0.99		
2b	2b-06	0.86		
2b	2b-07	0.98		
2b	2b-08	0.50		
2b	2b-09	1.00		
2b	2b-10	0.85		
2c	2c-01	1.00		
2c	2c-02	1.00		
2c	2c-03	1.00		
2c	2c-04	1.00		
2c	2c-05	1.00		
2c	2c-06	1.00		
2c	2c-07	1.00		
2c	2c-08	1.00		
2c	2c-09	1.00		
2c	2c-10	1.00		
2d	2d-01	1.00		
2d	2d-02	1.00		
2d	2d-03	1.00		

2d	2d-04	1.00		
2d	2d-05	1.00		
2d	2d-06	1.00		
2d	2d-07	1.00		
2d	2d-08	1.00		
2d	2d-09	1.00		
2d	2d-10	1.00		
2e	2e-01	0.80		
2e	2e-02	0.92		
2e	2e-03	**0.38**	**0.60**	**2g**
2e	2e-04	**0.08**	**0.87**	**2b**
2e	2e-05	0.96		
2e	2e-06	0.76		
2e	2e-07	**0.37**	**0.61**	**2g**
2e	2e-08	**0.08**	**0.91**	**2b**
2e	2e-09	0.96		
2f	2f-02	0.76		
2f	2f-03	0.69		
2f	2f-04	**0.27**	**0.69**	**2g**
2f	2f-05	0.95		
2f	2f-06	0.62		
2f	2f-07	0.54		
2f	2f-08	**0.12**	**0.83**	**2g**
2f	2f-10	0.90		
2g	2g-01	0.99		
2g	2g-02	0.67		
2g	2g-03	0.88		
2g	2g-04	0.96		
2g	2g-05	0.98		
2g	2g-06	**0.29**	**0.71**	**2e**
2g	2g-07	0.95		
2g	2g-08	**0.48**	**0.51**	**2e**
2g	2g-09	0.68		
2g	2g-10	**0.18**	**0.73**	**2f**
2h	2h-01	0.84		
2h	2h-02	0.55		
2h	2h-03	0.62		
2h	2h-04	**0.18**	**0.82**	**2a**

2h	2h-05	0.90		
2h	2h-06	0.91		
2h	2h-07	**0.35**	**0.62**	**2f**
2h	2h-08	0.98		
2h	2h-09	0.92		
